# Outcomes and Morbidities in Low-Birth-Weight Neonates: A Retrospective Study From Western India

**DOI:** 10.7759/cureus.61981

**Published:** 2024-06-08

**Authors:** Nirali R Dhivar, Rohankumar Gandhi, Yogesh Murugan, Hetal Vora

**Affiliations:** 1 Paediatrics and Child Health, Gujarat Medical Education & Research Society, Gandhinagar, IND; 2 Community and Family Medicine, Shri M. P. Shah Government Medical College, Jamnagar, IND; 3 Family Medicine, Guru Gobind Singh Government Hospital, Jamnagar, IND; 4 Paediatrics, Smt. Nathiba Hargovandas Lakhmichand Municipal Medical College, Ahmedabad, IND

**Keywords:** india, growth, neurodevelopmental outcomes, neonatal morbidity, low birth weight

## Abstract

Background: Low birth weight (LBW) increases infant morbidity and mortality and is a major public health concern, especially in resource-constrained settings. The purpose of this retrospective study was to assess the outcomes and morbidities related to LBW neonates referred to a neonatal intensive care unit (NICU) in Western India.

Methods: The present study examined the medical records of newborns weighing less than 2 kg at birth who were admitted to the NICU between September 15, 2016, and September 15, 2017. Data on long-term outcomes, clinical manifestations, morbidities, mortality, and demographic variables were gathered and analyzed. Descriptive statistics were used to present continuous variables as mean and standard deviation (SD), while categorical variables were presented as frequencies and percentages. Bivariate and multivariate logistic regression analyses were carried out to find the association between gestational age and major morbidities among LBW babies.

Results: Of 4710 births, 327 (6.9%) were LBW. The leading morbidities of LBW babies were respiratory distress syndrome (RDS) 153 (46.8%), neonatal jaundice 92 (28%), and septicemia 81 (25%), contributing to 58 (17.7%) deaths. Lower gestational age was associated with significantly higher adjusted odds of RDS (<28 weeks: reference; 28-32 weeks: adjusted odds ratio (AOR) 0.07, 95% confidence interval (CI) 0.01-0.33; ≥37 weeks: AOR 0.001, 95% CI 0.00005-0.02) and RDS-related mortality (28-32 weeks: AOR 0.26, 95% CI 0.06-1.13; ≥37 weeks: AOR 0.07, 95% CI 0.01-0.43). Among 250 successfully discharged cases, at 12 months, 18 (13.7%) had a weight below the 3rd percentile, and 9 (6.8%) failed the neurodevelopmental screening.

Conclusion: LBW infants in this setting experience significant morbidities, mortality, and long-term growth and developmental effects. To alleviate the burden associated with LBW, improved neonatal care facilities, infection control protocols, and focused interventions are essential.

## Introduction

Low birth weight (LBW) is a major public health concern globally, affecting around 15-20% of all births worldwide, with the highest prevalence seen in low- and middle-income countries [1, 2]. LBW refers to infants weighing less than 2500 g at delivery, and this group can be further categorized into very low birth weight (VLBW, <1500 g) and extremely low birth weight (ELBW, <1000 g) [3]. Neonates born with LBW are at an increased risk of numerous immediate and long-term complications, including sepsis, respiratory distress syndrome, necrotizing enterocolitis, intraventricular hemorrhage, and neurodevelopmental deficits [4, 5].

In India, the prevalence of LBW ranges from 15% to 30%, with notable regional variations [6, 7]. Despite advancements in neonatal care, LBW remains a significant contributor to infant mortality and morbidity in the country, accounting for 60-80% of all neonatal deaths nationwide [8, 9]. Furthermore, survivors of LBW often face long-term consequences such as stunting, cognitive and neurodevelopmental delays, and an increased risk of developing chronic conditions later in life [10, 11].

The considerable global burden of LBW, coupled with its adverse short- and long-term impacts, emphasizes the pressing need for improved understanding and management of this condition, particularly in resource-limited settings. While several studies have investigated the outcomes of LBW neonates in India, there is a paucity of comprehensive data from the western region of the country.

This study aimed to address this gap by evaluating the outcomes and morbidities associated with LBW neonates admitted to a tertiary care facility's Neonatal Intensive Care Unit (NICU) in Western India. The primary objectives were to investigate neonatal morbidities, mortality rates, and factors influencing survival, and to assess the developmental and growth outcomes among surviving infants during their first year of life. By examining the morbidities, causes of mortality, and long-term outcomes, this study sought to contribute to the existing knowledge base and guide the management and prevention of complications related to LBW in resource-constrained settings.

## Materials and methods

Study design and setting

The medical records of newborns hospitalized in the NICU of Smt. Shardaben Municipal General Hospital, Saraspur, Ahmedabad, Gujarat, India, between September 15, 2016, and September 15, 2017, were evaluated for this retrospective observational study.

Study population

All inborn newborns admitted to the NICU during the study period with a birth weight of <2.5 kg were included in the study population. Exclusion criteria included newborns weighing 2.5 kg or more at birth, out born neonates admitted to the NICU, and neonates with incomplete medical records lacking key data on gestational age, birth weight, clinical course, or outcomes.

Data collection

Various types of data collected from the medical records of neonates eligible for a study are presented in Table 1.

**Table 1:** Various Types of Data Collected From the Medical Records of Neonates Eligible for a Study SNCU: special newborn care unit, AGA: appropriate for gestational age, SGA: small for gestational age, LGA: large for gestational age, NICU: neonatal intensive care unit, MAS: meconium aspiration syndrome, RDS: respiratory distress syndrome, TTNB: transient tachypnea of the newborn, ARF: acute renal failure, IVH: intraventricular hemorrhage, DIC: disseminated intravascular coagulation, CHD: congenital heart disease, CSF: cerebrospinal fluid, 2D: two-dimensional, LAMA: left against medical advice, CPAP: continuous positive airway pressure, KMC: kangaroo mother care, OAE: otoacoustic emissions, ROP: retinopathy of prematurity, TDSC: Trivandrum developmental screening chart, IAP: Indian Academy of Paediatrics.

Category	Data
Demographic information	Full name, gender, SNCU number, address, date of birth, date of admission, age at admission (in hours/days)
Anthropometric measurements	Birth weight (in kg, measured using a digital weight scale with an accuracy of 0.001 g), gestational age (determined from the last menstrual period, first-trimester ultrasound, or Ballard score), classification (AGA, SGA, LGA)
Clinical data	Duration of NICU stay (categorized as <3 days, 3–6 days, 7–14 days, >14 days), risk factors, complications (prematurity, SGA, polycythemia, apnea, MAS, RDS, TTNB, pneumothorax, pneumonia, birth asphyxia, neonatal sepsis, meningitis, seizures, neonatal jaundice, multiple pregnancy, ARF, shock, IVH, hypothermia, neonatal hyperglycemia/hypoglycemia, hypocalcemia, DIC, congenital malformations, neonatal cardiac failure, neonatal diarrhea, CHD)
Growth assessment	Fenton growth charts used to assess growth until 40 weeks of gestational age
Investigations	CSF analysis, septic screening, neuroimaging, abdominal and cranial ultrasound, 2D echocardiography (as indicated by the patient's condition and disease process)
Discharge details	Immediate outcome at discharge (discharge, LAMA, expired, referred), discharge counseling (feeding, hygiene, warm care, danger signs), date of discharge, weight at discharge (measured using a digital scale with an accuracy of 0.001 g), head circumference, final diagnosis, specific treatments received (ventilatory care, CPAP, oxygen, phototherapy, surfactant, blood products, antibiotics, partial exchange transfusion, inotropes, feeding, supplements), duration of KMC, cranial and abdominal ultrasound findings, OAE screening, ROP screening (at 4 weeks of postnatal age or 32 weeks of postmenstrual age, whichever was later)
Follow-up data	Morbidity and mortality, vaccination status, supplements received, feeding method (breastfeeding or formula feeding), special remarks, developmental screening (using TDSC), neuromuscular tone (using Amiel-Tison angle: normal tone, hypotonia, hypertonia), referral to neurology clinic for abnormal screening results, growth monitoring (using IAP charts for term infants and Fenton charts for preterm infants until 40 weeks of corrected gestational age, then IAP charts), follow-up visits scheduled at discharge, contact via telephone for follow-up, documentation of recurrent admission and mortality causes after discharge for infants who attended follow-up visits

Data analysis

IBM SPSS Statistics for Windows, Version 26 (Released 2019; IBM Corp., Armonk, New York) was used to compile and analyze the data that had been collected. The analysis of the outcome, clinical, and demographic data was done using descriptive statistics. To determine the significance of the observed differences and to appraise the relationships between the variables, chi-square statistical tests and logistic regression analysis were used.

Ethical considerations

Ethical rules and principles guided the conduct of the investigation. Appropriate steps were taken to protect patient data privacy and confidentiality. The study was approved by the Institutional Ethics Committee of Smt. Shardaben Municipal General Hospital, Saraspur, Ahmedabad (Approval No. SMGH/IEC/2023/045).

## Results

An overview of the study population and results is shown in Table 2. Of the 327 LBW babies (6.9%), there were 105 intrauterine deaths (2.2%), resulting in a male-to-female ratio of 51.6% to 48.3%. Out of the infants with LBW, 250 (76.4%) were successfully discharged from the hospital, 58 (17.7%) expired in the intensive care unit, 16 (4.8%) were defined as left against medical advice (LAMA), and 3 (1%) were referred.

**Table 2:** Demographic and Outcome Data of Low-Birth-Weight Babies The data are represented as frequencies and percentages. IUD: intrauterine deaths, LBW: low birth weight, NICU: neonatal intensive care unit, LAMA: left against medical advice.

Parameters	Frequencies (%)
Total deliveries	4710
IUD	105 (2.2%)
Low birth weight babies (<2.5 kg)	327 (6.9%)
Male:female ratio	169 (51.6%):158 (48.4%)
Outcomes of LBW babies, N=327	
Successful discharge	250 (76.4%)
Expired in NICU	58 (17.7%)
Left against medical advice (LAMA)	16 (4.8%)
Referred	3 (1%)

Based on their birth weight and gestational age, the LBW newborns are categorized in Table 3. Of the 327 babies born with LBW, 85 (25.9%) were born with VLBW, and 16 (4.8%) were born with ELBW. In terms of gestational age, 78 (23.8%) babies were born between 28 and 32 weeks, 166 (50.7%) babies were born between 33 and 36 weeks, and 79 (24.1%) babies were born after 37 weeks. Four babies (1.2%) were born before 28 weeks. The distribution of gender within each gestational age group is also included in the table.

**Table 3:** Birth Weight and Gestational Age Distribution, N=327 The data are represented as frequencies and percentages.

Birth Weight	Frequencies (%)
Very low birth weight	85 (25.9%)
Extremely low birth weight	16 (4.8%)
Gestational age	
<28 weeks	4 (1.2%)
28-32 weeks	78 (23.8%)
33-36 weeks	166 (50.7%)
>37 weeks	79 (24.1%)

The LBW newborns are grouped in Table 4 into two categories: small for gestational age (SGA) and appropriate for gestational age (AGA), which are further subdivided into preterm and full-term groups. Preterm AGA made up 71 (21.7%) of the total, preterm SGA made up 182 (55.6%), and full-term SGA made up 74 (22.6%). The gender breakdown within each group is also shown in the table.

**Table 4:** Distribution of LBW Babies Based on AGA and SGA, N=327 The data are represented as frequencies and percentages. LBW: low birth weight, AGA: appropriate for gestational age, SGA: small for gestational age.

Category	Male	Female	Total
Preterm AGA	37 (52%)	34 (47.8%)	71 (21.7%)
Preterm SGA	89 (48.9%)	93 (51%)	182 (55.6%)
Full-term SGA	48 (64.8%)	26 (35%)	74 (22.6%)

Table 5 presents the results of bivariate and multivariate logistic regression analyses examining the association between gestational age and major morbidities among LBW babies.

**Table 5:** Association Between Gestational Age and Major Morbidities/Mortality Causes in LBW Babies A p-value of <0.05 is considered significant, and a p-value of <0.001 is considered highly significant. LBW: low birth weight, COR: crude odds ratio, AOR: adjusted odds ratio, adjusted for potential confounders such as maternal age, BMI, antenatal care, antepartum hemorrhage, pregnancy-induced hypertension, and maternal anemia.

Morbidity/Mortality	Gestational Age	Bivariate Analysis COR (95% CI)	p-Value	Multivariate Analysis, AOR (95% CI)	p-Value
Respiratory distress syndrome (RDS)	<28 weeks	Ref	-	Ref	-
	28-32 weeks	0.04 (0.01-0.18)	<0.001	0.07 (0.01-0.33)	0.001
	33-36 weeks	0.01 (0.002-0.03)	<0.001	0.02 (0.004-0.09)	<0.001
	≥37 weeks	0.0004 (0.00003-0.005)	<0.001	0.001 (0.00005-0.02)	<0.001
Neonatal jaundice	<28 weeks	Ref	-	Ref	-
	28-32 weeks	0.17 (0.02-1.33)	0.092	0.30 (0.03-2.90)	0.299
	33-36 weeks	0.03 (0.004-0.27)	0.001	0.07 (0.01-0.66)	0.019
	≥37 weeks	0.05 (0.01-0.45)	0.008	0.15 (0.02-1.39)	0.095
Septicemia	<28 weeks	Ref	-	Ref	-
	28-32 weeks	0.24 (0.03-2.25)	0.214	0.34 (0.03-3.53)	0.364
	33-36 weeks	0.32 (0.04-2.71)	0.294	0.49 (0.05-4.66)	0.538
	≥37 weeks	0.43 (0.05-3.61)	0.436	0.83 (0.08-8.29)	0.875
Mortality: RDS	<28 weeks	Ref	-	Ref	-
	28-32 weeks	0.19 (0.05-0.73)	0.016	0.26 (0.06-1.13)	0.072
	33-36 weeks	0.03 (0.01-0.13)	<0.001	0.05 (0.01-0.26)	<0.001
	≥37 weeks	0.03 (0.01-0.16)	<0.001	0.07 (0.01-0.43)	0.004
Mortality: Septicemia (without RDS)	<28 weeks	Ref	-	Ref	-
	28-32 weeks	0.12 (0.01-1.23)	0.073	0.17 (0.02-1.89)	0.149
	33-36 weeks	0.04 (0.004-0.40)	0.006	0.06 (0.01-0.66)	0.021
	≥37 weeks	0.20 (0.02-1.84)	0.155	0.36 (0.04-3.77)	0.395

The fourth column shows the adjusted odds ratios (AORs) and 95% CIs from the multivariate analysis, adjusted for potential confounders such as maternal age, BMI, antenatal care, antepartum hemorrhage, pregnancy-induced hypertension, and maternal anemia. The leading morbidities were respiratory distress syndrome (RDS), 153 (46.8%); neonatal jaundice, 92 (28%); and septicemia, 81 (25%).

For RDS, both the bivariate and multivariate analyses reveal a statistically significant trend, with lower gestational ages associated with higher odds compared to the less than 28-week reference group. Extremely preterm babies (≥37 weeks) had the lowest adjusted odds (AOR 0.001, 95% CI 0.00005-0.02).

A similar pattern is observed for neonatal jaundice in the bivariate analysis, although the association is not statistically significant in the multivariate model after adjusting for confounders. The odds of septicemia did not differ significantly between gestational age groups in either analysis.

Regarding mortality, babies born at lower gestational ages had significantly higher odds of RDS-related mortality compared to less than 28 weeks in both bivariate (28-32 weeks: OR 0.19, 95% CI 0.05-0.73; ≥37 weeks: OR 0.03, 95% CI 0.01-0.16) and multivariate analyses (28-32 weeks: AOR 0.26, 95% CI 0.06-1.13; ≥37 weeks: AOR 0.07, 95% CI 0.01-0.43).

For septicemia-related mortality without RDS, the bivariate analysis showed lower odds for 33-36 weeks (OR 0.04, 95% CI 0.004-0.40) and ≥37 weeks (OR 0.20, 95% CI 0.02-1.84) compared to less than 28 weeks, but this association was only statistically significant for the 33-36-week group in the multivariate model (AOR 0.06, 95% CI 0.01-0.66).

The developmental and growth outcomes of the LBW babies who were monitored after discharge are listed in Table 6. Of the 250 babies that were discharged successfully, 131 (40%) were lost to follow-up, 98 (30%) were followed up with, and 21 (6.4%) passed away within a year. The percentage of babies below the third centile for weight, height, and head circumference represents the catch-up growth at 12 months in the table. The table displays the neurodevelopmental outcomes in terms of the number of instances with aberrant otoacoustic emissions (OAE) and retinopathy of prematurity (ROP), as well as the percentage of children with abnormal neuromotor assessment and results from the Trivandrum Developmental Screening Chart (TDSC).

**Table 6:** Developmental and Growth Outcomes The data are represented as frequencies and percentages.

Follow-up Data	Frequencies (%)
Total successfully discharged	250
Followed-up	131 (40%)
Lost to follow-up	98 (30%)
Expired within 1 year	21 (6.4%)
Catch-up growth at 12 months (n=131)	
Weight < 3rd percentile	18 (13.7%)
Height < 3rd percentile	10 (7.7%)
Head circumference < 3rd percentile	11 (8.3%)
Neurodevelopmental outcome (n=131)	
Trivandrum Developmental Screening Chart (TDSC)	
Pass	122 (93.2%)
Fail	9 (6.8%)
Abnormal neuromotor assessment	5 (3.8%)
Retinopathy of prematurity (ROP)	4 (3%)
Abnormal otoacoustic emissions (OAE)	2 (1.5%)

## Discussion

This retrospective observational study provides crucial new insights into the long-term outcomes, morbidities, and mortality of LBW neonates hospitalized in the NICU. It was carried out at a tertiary care center in western India. The results of this study demonstrate the considerable difficulties in managing LBW newborns and emphasize the necessity of enhancing neonatal care services, especially in circumstances with low resources.

Of the 4,710 newborns born during the study period, 327 (6.9%) weighed less than 2.5 kg, which is in line with the 15% to 30% reported prevalence of LBW in India, likely reflecting socioeconomic and regional disparities [1, 6]. The study found that 55.6% (182 out of 327) of LBW newborns were SGA and preterm, while 22.6% (74 out of 327) were full-term SGA. These findings are consistent with the high prevalence of intrauterine growth restriction (IUGR) in this population, attributed to factors like maternal undernutrition, anemia, and inadequate antenatal care. The elevated risk is further compounded by the high prevalence of these risk factors among pregnant women in resource-poor settings [12].

The study found that among LBW neonates admitted to the NICU, there was a significant mortality rate of 17.7%, which is greater than the national average for the same period recorded by the Special Newborn Care Unit (SNCU) online data (UNICEF) [13].

RDS and septicemia accounted for 153 (46.8%) and 81 (25%) of morbidities, respectively, and were the primary causes of mortality. These results are consistent with earlier research that found sepsis and RDS to be the main causes of neonatal mortality in LBW babies [14, 15].

RDS and septicemia emerged as the leading causes of morbidity and mortality, which is unsurprising given the physiological vulnerability of LBW infants, particularly preterm ones. The significant association between lower gestational age and increased RDS risk/mortality can be explained by the developmental immaturity of the lungs and surfactant deficiency in preterm neonates [16]. Optimal thermal regulation and early surfactant administration may help mitigate this risk.

While septicemia rates did not differ significantly by gestational age, the overall burden (25%) highlights the need for stringent infection control protocols in NICUs. The high risk of sepsis in LBW neonates is multifactorial, involving immature immune systems, invasive procedures, and lapses in aseptic precautions during hospital stays [17].

Improved survival rates have been attributed to the use of inotropic medications, ventilatory support, and continuous positive airway pressure (CPAP), especially in infants delivered between 33 and 36 weeks' gestation. Extremely preterm infants (less than 28 weeks) had a much lower survival rate, emphasizing the necessity for sophisticated neonatal care facilities and interventions for this high-risk group [18].

The lack of association between gestational age and septicemia-related mortality could potentially be due to the early administration of appropriate antibiotics and supportive care, minimizing the impact of prematurity once sepsis develops. However, larger multicenter studies are needed to validate this observation.

Regarding long-term outcomes, the study found that a significant proportion of LBW infants experienced suboptimal growth and neurodevelopmental delays during the first year of life. At the age of one year, 18 out of 131 (13.7%) babies were below the third percentile for weight, 10 out of 131 (7.7%) were below the third percentile for height, and 11 out of 131 (8.3%) had a head circumference below the third percentile. These results are in line with earlier research that found LBW newborns had a higher chance of developmental delays and growth impairment [10, 11].

In terms of neurodevelopmental assessment using the TDSC, 3.8% (5 out of 131) of newborns had abnormal neuromotor evaluations, and 6.8% (9 out of 131) of infants failed the screening test. LBW infants are at an increased risk of long-term neurodevelopmental consequences, necessitating close monitoring and early intervention, even though the study did not find a statistically significant association between abnormal neurodevelopmental outcomes and factors such as gestational age or SGA status [19, 20].

Strengths and limitations

This study's key strengths include the comprehensive data on morbidities, mortality, and long-term outcomes, which are often lacking in LBW studies from resource-limited settings. However, the retrospective single-center design and possible bias from incomplete records limit the generalizability of findings. Future large-scale, prospective multicenter studies are warranted.

Recommendations

Strengthening antenatal care services and maternal nutrition interventions is crucial, given the high prevalence of preterm and SGA babies. Improved access to antenatal care and maternal nutrition programs, along with early identification and management of factors like maternal undernutrition, anemia, and pregnancy-related complications, could help reduce the incidence of LBW.

Enhancing neonatal care facilities and protocols for preterm and LBW infants is essential. Dedicated neonatal intensive care units with advanced equipment and specialized staff training are necessary to address the significant morbidity and mortality burden associated with prematurity and LBW. Implementing evidence-based protocols for respiratory care, thermoregulation, nutritional support, and infection control is vital.

Prioritizing early screening and management of RDS is important, as RDS is the leading cause of mortality, particularly in preterm infants. Strategies for early screening, prophylactic surfactant administration, and optimal respiratory management should be established. Functional regionalized perinatal care systems could facilitate timely transport and management of high-risk preterm deliveries.

Reinforcing infection prevention and control measures is critical, given the substantial contribution of septicemia to morbidity and mortality. Adherence to infection control protocols, judicious use of antibiotics, and continuous surveillance for antimicrobial resistance are essential in neonatal units.

Implementing comprehensive follow-up and early intervention programs is necessary to address the suboptimal growth and neurodevelopmental outcomes observed among LBW survivors. Structured follow-up programs should include growth monitoring, neurodevelopmental screening, early intervention services, nutritional counseling, and family support systems.

Investing in research and data systems for LBW is crucial. Prospective multicenter studies with larger sample sizes are needed to further characterize the risk factors, morbidities, and long-term implications of LBW in the Indian context. Strengthening data collection and surveillance systems can guide targeted interventions and resource allocation.

Adopting a multidisciplinary, collaborative approach is vital. Addressing the multifaceted challenges of LBW requires coordinated efforts involving obstetricians, neonatologists, pediatricians, nurses, public health professionals, policymakers, and the community. Fostering collaboration and knowledge-sharing across disciplines and institutions is essential for sustainable progress.

## Conclusions

This study emphasizes the substantial burden of long-term effects, morbidities, and mortality linked to LBW neonates in resource-constrained environments. The results highlight the necessity of better newborn care facilities, more stringent infection control protocols, and focused treatments to address maternal risk factors and support the best possible growth and neurodevelopmental outcomes for infants born before full term. Future studies should concentrate on developing and implementing evidence-based plans to lower the prevalence of LBW and enhance overall outcomes for this susceptible group.
